# Anakinra in Paediatric Rheumatology and Periodic Fever Clinics: Is the Higher Dose Safe?

**DOI:** 10.3389/fped.2022.823847

**Published:** 2022-03-07

**Authors:** Šárka Fingerhutová, Eva Jančová, Pavla Doležalová

**Affiliations:** ^1^Centre for Paediatric Rheumatology and Autoinflammatory Diseases, Department of Paediatrics and Inherited Metabolic Disorders, First Faculty of Medicine, Charles University and General University Hospital, Prague, Czechia; ^2^Department of Nephrology, First Faculty of Medicine, Charles University and General University Hospital, Prague, Czechia

**Keywords:** anakinra, safety, off-label anakinra, systemic juvenile arthritis, macrophage activation syndrome, autoinflammatory diseases (AID)

## Abstract

**Objective:**

Anakinra has been increasingly used in off-label indications as well as dosing and mode of administration in a variety of inflammatory conditions. We aimed to review our clinical practice and compare treatment outcomes with published data.

**Methods:**

Clinical data from electronic records were retrospectively reviewed for patients treated with anakinra over the past 6 years for autoinflammatory diseases (AID).

**Results:**

From 47 eligible patients (27 female patients), 32 were children. Macrophage activation syndrome (MAS) was the indication for anakinra therapy in 42.6% of patients. Systemic juvenile idiopathic arthritis (SJIA) was the most common underlying diagnosis (19/47) followed by the spectrum of AID. Off-label use was noted in 38.3% patients. Recommended dose was exceeded in 21 children (mean induction dose 5.1, highest dose 29.4 mg/kg/day) and two adults; five patients were treated intravenously. The mean treatment duration for SJIA was 1.4 years, that for AID was 2.2 years, and that for patients with higher anakinra dose was 9.7 (19.3) months. The mean follow-up duration was 2.7 (1.7) years. Treatment was effective in the majority of SJIA and cryopyrinopathy patients as well as those with MAS. Anakinra was well-tolerated without any major adverse effects even in patients with long-term administration of higher than recommended doses including two infants treated with a dose of over 20 mg/kg/day.

**Conclusion:**

Our results support early use of anakinra in the individually tailored dosing. In patients with hyperinflammation, anakinra may be lifesaving and may even allow for corticosteroid avoidance. Further studies are needed in order to set up generally accepted response parameters and define condition-specific optimal dosing regimen.

## Introduction

Anakinra, a biosynthetic analog of interleukin-1 (IL-1) receptor antagonist, increases its natural downregulating action of blocking the two isoforms of IL-1 from binding to their receptor. Resulting reduction of proinflammatory signaling has major therapeutic implications in a wide variety of diseases where dysregulation of innate immune mechanisms leads to IL-1 overproduction (1–3). The term “autoinflammation” has been implied to distinguish this phenomenon from autoimmunity, although the two mechanisms may co-exist, creating a pathophysiological continuum (2). Among systemic inflammatory conditions, mainly periodic fever syndromes and systemic juvenile idiopathic arthritis (SJIA)/adult-onset Still's disease (AOSD) may benefit from IL1 blockade (4–6). Although, European Medicines Agency (EMA)-approved indications of anakinra currently include apart from rheumatoid arthritis only cryopyrin-associated periodic syndromes (cryopyrinopathy, CAPS), familial Mediterranean fever (FMF), and SJIA/AOSD (7), its off-label use in other conditions has been demonstrated (8–13). Among others, anakinra has been successfully used in hyperinflammatory conditions associated with cytokine storm including macrophage-activation syndrome (MAS), severe COVID-19, as well as paediatric inflammatory multisystem syndrome temporarily associated with SARS-CoV-2 infection (PIMS-TS) (14–23).

Moreover, in diagnostically unconfirmed conditions, IL-1-mediated disease is highly suggestive when acute inflammation is rapidly withheld often within hours from the first administration of anakinra (24). Its short half-life and rapid therapeutic response when used appropriately allow its use as a diagnostic/therapeutic test (25). Not only indications but also anakinra dosing often deviates from its official label of 1–2 (maximum 4) mg/kg/day (children above 8 months of age and more than 10 kg of weight) or 100 mg/day in children >50 kg of weight and adults (7, 8, 17, 23, 24, 26).

Increasing and often off-label use of anakinra over the past decade has prompted us to review and analyze our own practice. The main aim of this study was to report the data on efficacy, safety, and tolerance of anakinra used in variable dosing regimens in patients with the spectrum of hyperinflammatory conditions.

## Patients and Methods

### Patient Selection

Patients attending fever clinics at the Center for Paediatric Rheumatology and Autoinflammatory Diseases (children and young adults) and Department of Nephrology (adult patients), General University Hospital in Prague were eligible for study participation. All patients in whom anakinra therapy was initiated between January 2015 and May 2021 for the diagnosis of periodic fever syndrome, SJIA, or other inflammatory condition suspected of IL-1 mediated disease were included. Additional inclusion criteria were follow-up duration of a minimum of 3 months from anakinra treatment onset and patient/legal guardian informed consent with study participation.

### Collected Data

Electronic hospital records were reviewed retrospectively. The baseline characteristics included patient demography, medical history, diagnostic procedures, laboratory results, and clinical presentation that led to anakinra therapy. Details on clinical response, anakinra dose and treatment duration, reason for discontinuation, adverse events, and need for concomitant therapies were retrieved.

### Therapeutic Response Definition

Initial therapeutic response was assessed within 1 week of treatment and was defined as full, partial, or no response. Complete regression of disease signs with normalization of inflammatory parameters without the need for corticosteroids was graded as full response. Patients who responded to anakinra but required concomitant corticosteroids were considered partial responders as did those who showed either incomplete clinical improvement or retained residual inflammation in their laboratory parameters. Long-term efficacy was assessed by achievement of disease inactivity. For the purpose of this study, inactive disease was defined as a full absence of laboratory inflammation and clinical disease signs together with the lowest possible score (zero) in the physician global assessment (PGA) of disease activity (recorded for SJIA) or Autoinflammatory Diseases Activity Index (AIDAI) recorded by patients/parents (recorded for periodic fever syndromes) and no concomitant corticosteroid therapy. Main laboratory parameters of inflammation included erythrocyte sedimentation rate (ESR), white blood cell count (WBC), C-reactive protein (CRP), and serum amyloid A (SAA).

### Statistical Analysis

Data are expressed as mean ± standard deviation (SD) for interval and ordinal variables and counts with percentages for categorical variables.

## Results

### Patient Characteristics

From the total of 60 patients who were prescribed anakinra at our unit, 13 were excluded from analysis because they either started the treatment prior to inclusion date (*n* = 3) or were not followed up for sufficient time (*n* = 10). From 47 patients (27 female patients) who fulfilled inclusion criteria, 32 (68.1%) were children (17 girls). All but one Asian boy with SJIA were Caucasians. Main demographic data and diagnoses of patients in both paediatric and adult cohort are summarized in Table 1. The mean age at anakinra treatment onset in children and adults was 8.9 (SD 5.7) and 33.8 (SD 12) years, respectively. The two youngest patients were infants 8 and 33 days old weighing 2,300 and 2,950 g, respectively. In general, MAS was the most frequent reason for anakinra therapy (*n* = 20/47, 42.6%). It was a presenting feature of the underlying condition in seven patients [six SJIA, one Syndrome of Undifferentiated Recurrent Fevers (SURF)]. MAS in SJIA accounted for 15/20 MAS patients; in three cases, MAS complicated SURF and two patients had other conditions—one polyarticular JIA and one NLR Family CARD Domain Containing 4-associated AID (NLRC4-AID) (Table 1).

**Table 1:** Main patient characteristics.

**Diagnosis**		**Number (** ***N*** **) of patients**		**Age in years (SD) at**			**Duration (SD) of**			**Initial response to anakinra therapy, *N* (%)^&^**	
		***N* (Adult)**	**Female, *N* (%)**	**Disease onset**	**Diagnosis**	**Starting anakinra**	**Disease before anakinra**	**Anakinra therapy**	Follow ^*^	**Full**	**Partial**
SJIA	With MAS	15 (1)	9 (60)	8 (4.6)	8.1 (4.6)	10 (5)	19.1 (13.9) days	1.4 (1)	2.6 (1.1)	8 (53.3)	7 (46.7)
	Without MAS	4 (0)	3 (75)	2.7 (1.3)	2.9 (1.3)	4.6 (3.5)	1.9 (3.4) years	1.1 (1.2)	1.6 (1.1)	2 (50)	2 (50)
CAPS		10 (9)	6 (60)	7 (9.5)	32.1 (18.4)	34 (16.5)	27.4 (14.6) years	3.9 (1.8)	4 (2)	7 (70)	2 (20)
MKD		6 (4)	4 (66.7)	2.1 (2.2)	14.7 (9.5)	23 (11.3)	21.5 (12.8) years	2.1 (2.1)	2.5 (1.8)	0	6 (100)
SURF	With MAS	3 (1)	2 (66.7)	11.7 (8.7)	12.4 (9.5)	12.4 (9.5)	9.0 (14.7) months	1.2 (0.1)	1.9 (0.9)	2 (66.7)	1 (33.3)
	Without MAS	3 (0)	0	3.6 (2.3)	4 (2.5)	9.5 (6.1)	6 (4.7) years	0.2 (0.2)	1.3 (0.6)	2 (66.7)	0
PIMS-TS		2 (0)	0	13 (4)	13 (4)	13 (4)	7 (1.4) days	0.1 (0.04)	0.3 (0)	2 (100)	0
Other AID		2 (0)	2 (100)	3 (2.8)	6.6 (3.2)	14 (3)	11.7 (0.2) years	0.5 (0.3)	1.8 (1.5)	0	0
Other MAS		2 (0)	0	7.8 (7.8)	8.2 (7.8)	7.9 (7.8)	5.5 (7.8) years	3.3 (3.3)	5.2 (1.3)	1 (50)	1 (50)
ALL MAS		20 (2)	12 (60)	8.5 (5.9)	8.8 (6.1)	10.2 (6.3)	1.6 (5.9) months	1.5 (1.5)	2.7 (1.4)	11 (55)	9 (45)

*SJIA, systemic juvenile idiopathic arthritis; MAS, macrophage activation syndrome; CAPS, cryopyrin-associated periodic syndrome; MKD, mevalonate-kinase deficiency; SURF, Syndrome of Undifferentiated Recurrent Fever; PIMS-TS, Paediatric inflammatory multisystem syndrome temporarily associated with SARS-CoV2; AID, autoinflammatory diseases; Other MAS, NLRC4-associated autoinflammatory disease and polyarticular JIA; Other AID, Blau syndrome and Pyogenic arthritis, pyoderma gangrenosum and acne syndrome*.

* *Time from starting anakinra to the last follow-up*.

& *Initial therapeutic response assessed within 1 week of treatment. Full response, complete regression of disease signs with normalization of inflammatory parameters without the need for corticosteroids; partial response, incomplete clinical improvement or residual inflammation in laboratory parameters or concomitant corticosteroids*.

Late diagnosis of CAPS prevailed among indications of anakinra in adults (*n* = 9/15; 60%) followed by mevalonate-kinase deficiency (MKD) (*n* = 4/15; 26.7%). In children, SJIA with or without MAS was the main reason for anakinra treatment (*n* = 19/32; 59.4%) (Table 1). All SJIA patients fulfilled the International League of Associations for Rheumatology classification (27). MAS was diagnosed according to the published criteria (28). Diagnosis of monogenic autoinflammatory diseases was confirmed by Sanger sequencing. Diagnosis of the Syndrome of Undifferentiated Recurrent fever (SURF) in both children and adults was based on patient history of recurrent self-limited febrile episodes accompanied by systemic inflammation without proven infectious or genetic etiology, not fulfilling the criteria for periodic fever, aphthous stomatitis, pharyngitis, and adenopathy (PFAPA) syndrome (29). In summary, 18/47 (38.3%) patients received anakinra off-label. Mean duration of anakinra treatment until the last follow-up in August 2021 was 1.9 (SD 1.9) years. From anakinra initiation, patients were followed for the mean of 2.7 years (SD 1.7).

### Anakinra Administration

Majority of patients received anakinra as subcutaneous injections. In 17 patients who required more than 100 mg/day, the daily dose was divided into 2 (200 mg/day, 14 cases) or 3 (300 mg/day, 3 cases) separate 100-mg injections. Hospitalized patients who had a venous access for fluid or other drug administration received anakinra intravenously during the acute phase of the disease (*n* = 5) in order to delay painful subcutaneous administration until clinical improvement. Daily dose of anakinra was administered in 1–3 intravenous infusions (in 10–100 ml of saline, according to the dose and the weight of the patient). Continuous administration of intravenous anakinra was used for 4 days for the treatment of an adolescent patient with severe PIMS-TS with multiorgan involvement and myocarditis. Only one patient with MKD received on-demand anakinra during febrile episodes.

### Anakinra Dosing

Recommended dosing of anakinra (1–4 mg/kg/day) was exceeded on one or more occasions during the follow-up in 21 children and 2 adults. All adults initially received 100 mg of anakinra daily corresponding to the mean dose of 1.9 (SD 0.85) mg/kg. Four of them (3 CAPS, 1 MKD) required increase of their maintenance dose to 200 mg/day for the recurrence of disease activity while on 100 mg/day. One adult patient with SURF and chronic MAS required long-term administration of 300 mg of anakinra daily (4.9 mg/kg) in order to maintain clinically acceptable disease activity. Another obese adult patient with SJIA and recurrent MAS received 600 mg of anakinra daily (6.3 mg/kg) for 5 days in order to handle severe disease relapse with subsequent slow reduction to 300 mg/day (maintained for 15 months) and then to 200 mg/day (ongoing for 19 months).

**Figure 1:**
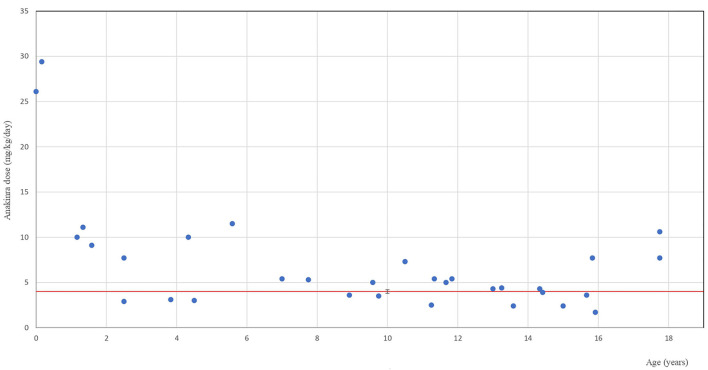
Anakinra therapy in children. The highest anakinra dose used for at least 3 weeks in individual paediatric patients in relation to their age. The blue dots indicate individual patients at the age when they received the highest anakinra dose. The red line indicates the recommended dose cut-off.

Throughout the follow-up, children received anakinra in a wide dose range of 1.4–29.4 mg/kg/day. Their mean induction dose was 5.1 (SD 4.18) mg/kg. It was chosen individually on the basis of patient age/weight and severity of the condition. Practical aspects of the prefilled injection fluid volume (0.67 ml/100 mg) were also taken into account with 0.2 ml (~30 mg) being the smallest individual dose. Children with chronic or recurrent conditions (SJIA and MKD) received the mean initial dose of 2.78 (SD 1.3) mg/kg/day. The dose was doubled in one SJIA patient after 2 weeks of therapy for the relapse of systemic symptoms. In 8/22 (36.4%) patients with acute severe conditions (20 MAS and 2 PIMS-TS) with insufficient response to the initial mean anakinra dose of 5.65 (SD 4.7) mg/kg/day within the first 24 h, it was then doubled or tripled within the next 2–4 days until clinical response was achieved. Figure 1 shows the highest doses used for the minimum duration of 3 weeks in individual patients in relation to their age with the red line indicating the recommended dose cut-off (4 mg/kg/day). Doses exceeding 4 mg/kg/day were administered for the mean treatment duration of 9.71 months (SD 19.3) to 21 patients. All five children who received high-dose anakinra (10–14.9 mg/kg, *n* = 3 and >15 mg/kg, *n* = 2) were small children (5 days to 5 years of age) suffering with the following conditions: SJIA ± MAS (*n* = 2), SURF + MAS (*n* = 1), chronic infantile neurological cutaneous and articular (CINCA) syndrome (*n* = 1), and NLRC4-AID (*n* = 1). An adolescent with severe PIMS-TS received continuous i.v. anakinra infusion at 10.6 mg/kg/day (total 792 mg/day). Anakinra diluted in saline (100 mg/10 ml) was administered at a rate of 3.3 ml/h for 4 days followed by subcutaneously administered 400 and 300 mg/day for an additional 1 and 2 weeks, respectively, with further dose reduction and withdrawal within 3 months.

### Therapy Outcomes

#### Systemic Juvenile Idiopathic Arthritis

From the SJIA cohort (*n* = 19), anakinra therapy was initiated in 15 (79%) patients due to a MAS episode. Anakinra was started after 19.1 (SD 13.9) days from the onset of fever that evolved into the MAS. MAS was the first manifestation of SJIA in six cases. In nine patients, MAS complicated SJIA after 50.6 (SD 49.4) months of the previous disease duration. Eight patients (8/15, 53.3%) responded fully without any corticosteroids and 7/15 (46.7%) partially. Only four patients received anakinra for active systemic disease without MAS. Two of them responded fully and two partially. In all SJIA patients, previous disease duration in full and partial responders was 5.1 (SD 11.6) and 44 (SD 50.2) months, respectively. Overall, in 10 full responders, systemic features disappeared within 1–2 days, and laboratory markers normalized within 2–16 weeks. Disease inactivity (off-steroids) was reached at a mean of 4.6 months (SD 6.1) in 11/19 (57.9%) patients (10 full responders who did not receive corticosteroids and 1 partial responder who initially needed corticosteroids). Anakinra was discontinued in 8 of them after a mean of 12.3 months (SD 5.1) of treatment, and they remained in drug-free remission until the last follow-up [mean follow-up 16.9 months (SD 9.1)]. In two preschool children, non-compliance to daily injections led to the switch to canakinumab after they reached disease inactivity with anakinra monotherapy. From nine partial responders, five remained on anakinra, and four patients were switched to another biologic.

#### Cryopyrin-Associated Periodic Syndromes

From eight patients with Muckle–Wells syndrome (MWS), seven responded fully within hours to days to standard anakinra dose from the first injection; only in one of them did the rash require 3 months of therapy to disappear. Impaired renal function due to AA amyloidosis in one patient remained stable as did audiometry in two patients with mild hearing loss. Two patients required dose escalation (to 200 mg/day) in order to maintain disease inactivity (30). One polymorbid adult patient died after 2 months of therapy for a pre-existing condition. In two patients with CINCA syndrome, anakinra was used for chronic meningitis after insufficient response to canakinumab. Nevertheless, even higher anakinra dose was only partially effective. All but one CAPS patients remained on anakinra therapy at the last follow-up.

#### Mevalonate Kinase Deficiency

All six patients with MKD had partial response to anakinra. The first injection administered at the beginning of an episode was able to terminate it in all patients, but subsequent daily treatment did not prevent disease recurrence. Although, all treated patients reported lower frequency of episodes on anakinra, their inflammatory activity between episodes persisted. In one adult, poor treatment adherence to the standard dose prevented its escalation. Two affected adult siblings refused further treatment and were lost to follow-up. In a 4-year-old child, episodic anakinra was ineffective, and non-compliance to subsequent administration of daily injections led to the switch to canakinumab. Therefore, only three patients remained on anakinra treatment at the last follow-up.

#### Syndrome of Undifferentiated Recurrent Fevers

From six patients with SURF, anakinra was indicated for MAS in three cases. Four children (including two with MAS) had complete response and two of them were able to discontinue anakinra after 14 and 16 months and remained in drug-free remission until the last follow-up (22 and 5 months). An adult patient with features of chronic MAS receiving 300 mg/day of anakinra partially responded but needed a switch to etoposide after 12 months due to persistently active MAS. One child did not respond despite receiving a high dose of anakinra (11.1 mg/kg/day) for 11 days and was switched to canakinumab.

#### PIMS-TS and Other Autoinflammatory Diseases

Patients treated for PIMS-TS had complete response to anakinra within days and were able to withdraw it within 3 months. An additional two patients with other autoinflammatory diseases (Blau syndrome and pyogenic arthritis, pyoderma gangrenosum and acne) failed to respond to anakinra. A patient with NLRC4-AID responded only partially to long-term therapy with high-dose anakinra. One child with polyarticular JIA in full remission and infection-triggered MAS had a full response to anakinra and was able to discontinue it in 2 weeks.

### Anakinra Tolerance and Adverse Events

The most frequent recorded adverse event (AE) was injection-site reaction in 27.7% of patients (seven adults and six children). It disappeared within 1 month in all patients. Eosinophilia was documented in 10/47 (21.3%) patients. It developed usually within the first 4–6 weeks, and in cases with mild elevation (0.5–2.0 × 10^9^/L), it disappeared spontaneously within 2 months. Higher eosinophilia with prolonged duration in 2 SJIA patients (3.6 and 2.3 × 10^9^/L) was associated with severe disease with lung involvement in one of them. Mild transient elevation of transaminases of up to 2 times the upper limit of normal (ULN) was noted in 4/47 (8.5%) patients and did not require any action. In one adolescent patient with SURF who developed hepatopathy with transaminases of 5–9 times ULN after 6 weeks on standard anakinra dose (1.9 mg/kg/day), transient interruption of therapy for 5 days led to the disease relapse as well as to normalization of transaminase levels. He responded well to the corticosteroid course and then to alternate-day anakinra with no further laboratory abnormality. Mild as respiratory infections have been reported in a similar frequency prior to anakinra therapy.

Overall, 5/47 (10.6%) patients who received anakinra experienced at least one severe adverse event (SAE). A 2-year-old infant with SJIA developed epilepsy while on anakinra for 5 months. One patient with SJIA was hospitalized due to a moderately severe COVID with transient need of increased anakinra dose. An adult patient (57 years old) with CAPS was hospitalized for total hip replacement surgery due to osteoarthritis. Another polymorbid adult patient with MWS died at 56 years of age after 2 months of anakinra for a relapse of pre-existing pancreatitis and acute pulmonary embolism. The girl with Blau syndrome had an episode of neurosarcoidosis requiring hospitalization and high-dose intravenous steroids. All SAE appeared in patients who had standard doses of anakinra.

## Discussion

This report on anakinra therapy uniquely combines details on dosing and mode of administration with data on short-term response as well as long-term treatment outcomes and side effects in children and adults with a spectrum of inflammatory conditions.

Besides larger studies of anakinra use in approved indications, there are multiple reports on its off-label use in a wide spectrum of chronic, recurrent, as well as acute inflammatory conditions (3, 25, 31, 32). The two largest multi-center studies retrospectively described the use of IL-1 blockers in both adults and children in France (9) and in Italy (13). Both studies reported high proportion of patients who received anakinra off-label [56.1% (9) and 85.7% (13), respectively]. Over the recent years, anakinra use in hyperinflammatory disorders has been more frequently reported with MAS/secondary hemophagocytic lymphohistiocytosis (sHLH) being the leading condition (16, 17, 23, 26, 33, 34). Even more recently, efficacy of anakinra in cytokine storm associated with COVID-19 or PIMS-TS was shown (16, 17, 35, 36). In our series, 20/47 patients received anakinra for features of MAS and nearly 40% were treated off-label.

Although, the two major published series reviewed larger patient population, they did not report use of higher than recommended dosing (13) or used it in a limited way of up to 6 mg/kg/day in some children only (9). In adults, the highest reported dose of anakinra administered intravenously in continuous infusion was 2 mg/kg/h for 72 h in the sepsis trial (37). In the MAS case series, it ranged between 2,400 and 3,500 mg/day and was used for several days with subsequent slow dose reduction upon clinical improvement (16). Although, the highest intravenous dose we used for 5 days in an adolescent patient of adult body size was nearly 800 mg/day, 2 other adult patients were treated with 300 mg of anakinra daily for over 1 year. To our knowledge, such a long-term use of supranormal anakinra dose for prolonged interval has not been reported in the literature.

Children often required higher doses of anakinra to achieve sustained disease remission (38). Intravenous administration was often used in hospitalized patients with severe disease and/or in order to avoid painful subcutaneous injections. High, often intravenous anakinra doses have been used mainly for severe conditions associated with MAS/sHLH (17). Doses higher than 10 mg/kg/day (maximum 20) were used in 6/19 mostly paediatric patients with MAS for up to 54 days (34). The highest reported dose in children was 48 mg/kg/day given for days to weeks in patients with non-familial central nervous system hemophagocytic lymphohistiocytosis and secondary MAS due to systemic lupus erythematosus or infection (26, 33). Administration of the dose up to 10 mg/kg/day was reported also in other conditions like Kawasaki disease (11, 39), febrile infection-related epilepsy syndrome disease (40), or CINCA syndrome (24). Five children from our cohort received high-dose anakinra (>10 mg/kg/day) with the highest dose of 26.1 and 29.4 mg/kg/day administered to infants 5 days and 2 months old, respectively, for more than 3 weeks.

Comparison of response to anakinra therapy with published studies is hampered by their different design and the lack of uniform outcome parameters. We have applied modification of existing definitions (9, 13, 31, 32) of complete, partial, and no response that we enriched by adding the absence of any corticosteroid therapy in order to qualify for the complete response. We have also used an arbitrary time frame for the evaluation of the initial response to anakinra as being up to 1 week of therapy. Using these definitions, our complete response rates were lower than reported by Vitale et al. (13) mainly for SJIA (52.6 vs. 86.36%), probably due to the higher proportion of Italian patients receiving concomitant corticosteroids. Nevertheless, all 10 complete responders out of 19 SJIA patients in our study were able to achieve clinical inactive disease by 6 months without use of any corticosteroids. This high rate of clinical response to anakinra in steroid-naïve SJIA patients is in agreement with the recent Dutch reports (41, 42). Full responders to anakinra had shorter previous disease duration than partial responders, an observation that is in agreement with the recent report on predictors of anakinra response (43). Anakinra use in MAS or other secondary HLH was recently reported by several groups (16–18, 33, 34, 44, 45). Despite different anakinra dosing, all of them reported its beneficial effect in majority of patients, although comparisons are difficult mainly due to variable concomitant corticosteroid use. From our 20 patients who received anakinra for MAS, 11 (55%) did not require additional corticosteroids and had complete response to anakinra monotherapy. In agreement with others, our data support anakinra use in urgent situations where flexible dosing due to its short half-life allows for its rapid adjustments in relation to the therapeutic response.

Adverse events observed in our patients were uncommon and similar to those reported in the literature with the self-limited skin reaction at the site of injection being the most frequent. Anakinra tolerance in children was limited by its painful application and lead to the switch to canakinumab despite its good therapeutic effect in five patients. We also paid attention to the occurrence of unexplained eosinophilia especially in patients with SJIA and lung disease, which was the case for one of our patients with eosinophilia (46). No adverse events except reversible rise of liver transaminases in just a few patients were attributed to high-dose anakinra (34).

## Conclusion

Our approach to anakinra therapy especially in acute or severe inflammatory conditions has evolved over the years from the strict adherence to recommended dosing and route of administration to a more flexible and patient-tailored regime. Instead of using anakinra only after insufficient response to corticosteroids, we now more often consider prescribing it as a first-line therapy. Moreover, a higher than recommended dose of anakinra often needed to achieve sufficient therapeutic response was well-tolerated without any significant adverse events. We believe that appropriate use of anakinra in terms of the dose and early timing may, in some patients, allow for rapid switching off the acute inflammation without the need for concomitant corticosteroids or at least without their long-term use.

## Data Availability Statement

The raw data supporting the conclusions of this article will be made available by the authors, without undue reservation.

## Ethics Statement

The studies involving human participants were reviewed and approved by Ethics Committee of the General University Hospital. Written informed consent to participate in this study was provided by the participants' legal guardian/next of kin.

## Author Contributions

ŠF and PD coordinated and performed most of the clinical analysis and drafted the manuscript. EJ contributed to clinical observations and patients follow-up. All authors contributed to conception, analysis and interpretation of data, and participated in revising the article and gave final approval of the version to be submitted.

## Funding

This study was supported by the Czech Health Research Council (AZV CR) grant NU21-05-00522.

## Conflict of Interest

The authors declare that the research was conducted in the absence of any commercial or financial relationships that could be construed as a potential conflict of interest.

## Publisher's Note

All claims expressed in this article are solely those of the authors and do not necessarily represent those of their affiliated organizations, or those of the publisher, the editors and the reviewers. Any product that may be evaluated in this article, or claim that may be made by its manufacturer, is not guaranteed or endorsed by the publisher.
